# Clinical course and neurological outcomes of cerebral venous sinus thrombosis: A single center retrospective observational study

**DOI:** 10.1371/journal.pone.0316849

**Published:** 2025-01-13

**Authors:** Pasook Sitthilok, Piangrawee Niprapan, Adisak Tantiworawit, Teerachat Punnachet, Nonthakorn Hantrakun, Pokpong Piriyakhuntorn, Thanawat Rattanathammethee, Sasinee Hantrakool, Ekarat Rattarittamrong, Lalita Norasetthada, Chatree Chai-Adisaksopha

**Affiliations:** 1 Department of Internal Medicine, Faculty of Medicine, Chiang Mai University, Chiang Mai, Thailand; 2 Division of Hematology, Department of Internal Medicine, Faculty of Medicine, Chiang Mai University, Chiang Mai, Thailand; BSMMU: Bangabandhu Sheikh Mujib Medical University, BANGLADESH

## Abstract

**Background:**

Cerebral venous sinus thrombosis (CVST) is a rare type of thrombosis that affects the cerebral venous system. The data on neurological outcomes are limited.

**Objectives:**

This study aimed to investigate the neurological outcomes of CVST, contributing factors, clinical presentation, treatment and mortality.

**Methods:**

This was a single-center, retrospective study at a university-based referral hospital in Thailand. Consecutive patients diagnosed with CVST between January 2010 and December 2020 were included. Outcomes were neurological outcomes measured by modified Rankin Scale (mRS), anticoagulant treatment, recurrence, and mortality.

**Results:**

One hundred and seven CVST patients were included with a mean age (± SD) of 42.7 ± 20.4 years. Following the treatment, neurological outcomes significantly improved, with the proportion of patients with mRS 0–1 increasing from 18.7% at diagnosis to 83.2% and 85.1% at three and six months, respectively (P < 0.001). Clinical variables were associated with residual neurological symptoms (mRS≥1) included age ≥ 50 years (OR 4.1, 95% CI; 1.7–9.4, P 0.001), male sex (OR 3.0, 95%CI; 1.4–6.6, P 0.006), the thrombus involvement in deep sinus system (OR 6.1, 95%CI; 1.1–76.9, P 0.04) and cerebral vein and dural sinus thrombosis (CVT) risk score ≥ 1 (OR 3.1, 95%CI; 1.3–7.5, P 0.014). Patients whose CVST was associated with hormonal therapy were found to have a lower risk of residual neurological symptoms (OR 0.2, 95%CI 0.1–0.6, P 0.004). Hormonal therapy was associated with anticoagulant discontinuation (OR 2.7, 95% CI 1.1–7.0; P = 0.04). The presence of malignancy increased the risk of bleeding (OR 5.8, 95% CI 1.4–24.1; P 0.016). Overall mortality was 2.8%. Of which 50% were related to major bleeding.

**Conclusions:**

A significant improvement in neurological outcomes was observed at 3 and 6 months after diagnosis. Older age, male sex, thrombus involvement in deep sinus system were associated with residual neurological symptoms.

## Introduction

Cerebral venous sinus thrombosis (CVST) is a type of thrombosis that occurs in the cerebral venous system and is categorized as an unusual site thrombosis. However, CVST has distinct contributing factors, severity, and prognosis compared to other unusual sites of thrombosis. CVST is known as a rare disease that has been reported more frequently in recent years. Previous studies reported an incidence rate of 0.2 to 1.6 per 100,000 population per year [1, 2]. However, the recent meta-analysis of the population-based studies revealed that the incidence of CVST increased over time. The overall incidence of CVST was measured at 8.7 per million [3].

In contrast to the arterial thrombosis, contributing factors of CVST encompass various elements. These include the use of oral contraceptive, pregnancy or post partum, hormone replacement therapy, head and neck infection, dehydration, malignancies, autoimmune conditions such as antiphospholipid syndrome or connective tissue diseaes, inherited thrombophilias, certain medications like L-asparaginase and mechanical injuries [4].

The mainstay treatment of CVST is an anticoagulant therapy. The European Stroke Organization and the American Heart Association recommended the use of low-molecular-weight heparin for the initial management of CVST, followed by transition to oral anticoagulant [4, 5]. Recent studies have shown that direct oral anticoagulants (DOACs) are as effective and safe as warfarin in treating CVST [6–9]. The neurological outcome is a critical factor in the management and treatment of CVST. Therefore, it is essential to investigate the neurological outcomes in patients with CVST, including factors contributing to neurological improvement or deterioration [10].

Comparing the neurological outcome of CVST with stroke, a previous study found that the rates of disability and mortality were lower in patients with CVST than in those with ischemic stroke particularly those who receive timely and appropriate treatment [11, 12]. However, some studies revealed mortality and disability up to 50% in CVST [13].

Due to substantial differences in contributing factors, clinical manifestations, treatments, and clinical outcomes, CVST stands apart from arterial thrombosis. Furthermore, there is a scarcity of data regarding the outcomes in patients affected by CVST. In this study, we aimed to investigate neurological outcomes of CVST measured by the modified Rankin Scale (mRS), a widely-used measure of neurological disability and dependence [14]. We examined all-cause mortality, bleeding complications, and recanalization rates.

## Materials and methods

### Study design

The study was a single-center, observational, retrospective cohort study with approval from the Research Ethics Board (No. 235/2564) at the Faculty of Medicine, Chiang Mai University, which was a university-based-referral hospital in Thailand. The data has been accessed from 10 July 2021 to 12 Jan 2022. The authors had no access to information that could identify individual participants during or after data collection.

### Patient population

#### Inclusion criteria

This study included consecutive patients aged ≥ 18 years who were newly diagnosed with CVST between January 2010 and December 2020. CVST was objectively diagnosed by contrast-enhanced computerized tomography (CT) scan, CT-venography, magnetic resonance imaging (MRI), or MR-venography. The radiologic diagnosis of CVST was based on the direct visualization of intraluminal venous thrombus in the venous sinus system or clear demonstration of absence of blood flow through CT or MRI imaging [4]. All imaging and diagnosis were reviewed by the investigators.

#### Exclusion criteria

Patients with incomplete data on critical variables, including neurological imaging, anticoagulant treatment regimen, and follow-up information were excluded from the study. Patients who were lost to follow-up before six months were excluded.

Patients with diagnoses other than CVST or repeat presentations of pre-existing CVST were excluded from the study.

### Demographic and clinical data

The following data were reviewed as follows: date of CVST diagnosis, imaging modality for diagnosis, site and involving veins, presenting symptoms and signs, neurological assessment using mRS, initial anticoagulant management, surgical intervention, long-term anticoagulant management, duration of anticoagulant treatment. The initial anticoagulant management was defined as the first 5–21 days of treatment [15]. The potential contribution factors were reviewed, including head and neck infection, head trauma, vascular abnormalities, hormonal therapy, cancer, hematologic malignancy, or myeloproliferative neoplasms. Patients who permanently discontinued anticoagulants were defined as "anticoagulant discontinuing," while those who continued anticoagulants until the last follow-up visit were defined as "anticoagulant continuing”.

We utilized the cerebral vein and dural sinus thrombosis (CVT) risk score proposed by the International Study on Cerebral Vein and Dural Sinus Thrombosis (ISCVT) [16]. The CVT risk score incorporates clinical variables including malignancy, coma, thrombosis of the deep venous system, mental status disturbance, male gender, and intracranial hemorrhage at presentation. The CVT risk score ranges from 0 to 9 with a higher score indicating a worse prognosis in terms of neurological outcomes. As per the original score, patients with a CVT risk score of ≥ 3 were classified as high-risk, and those with a score of < 3 were classified as low-risk. Death was confirmed through the patient database.

### Follow-up

The follow-up schedule was managed by treating physicians. According to local practice, CVST patients were scheduled for monitoring anticoagulant therapy and clinical follow-up every 1–3 months. We collected the clinical outcomes of patients at 3 months and 6 months, after diagnosis, as well as at subsequent follow-up visits until their last appointment. Recorded follow-up data were as follows: anticoagulant therapy, mRS, death, bleeding complication, recurrent CVST, and recanalization of CVST by neuroimaging. We followed all patients until the last date of follow-up visit or death.

### Outcomes

#### Primary outcomes

The primary outcome was the neurological outcome, as defined by mRS [17, 18]. Patients were classified into the following categories: no symptoms (mRS 0), no significant disability despite symptoms (mRS 1), slight disability (mRS 2), moderate to severe disability (mRS 3–5), or death (mRS 6) [17, 18]. We compared the changes of mRS at presentation, three months, and six months after diagnosis. The mRS was utilized as an ordinary scale. For the analysis of the primary outcome, we categorized the mRS into two groups: patients with no or mild disability (mRS 0–1) and patients with significant disability (mRS 2–6). The outcomes were compared at the time of presentation, and then at three and six months. Additionally, we conducted an analysis for those who had no residual symptoms (mRS < 1).

#### Secondary outcomes

The secondary outcomes were death as a separate outcome, bleeding complication, recurrent CVST, and recanalization of CVST. The recanalization of CVST was evaluated using neuroimaging (CT venography, MR venography, non-contrast CT, or MRI). Recanalization was defined as the regression of venous thrombus as compared to the initial imaging [19]. Recanalization was classified as complete, partial, stable, or progression. Bleeding complications were assessed and the site and severity of bleeding were described according to the International Society on Thrombosis and Hemostasis bleeding scale (ISTH) [20]. Recurrent CVST was defined as a new or progression of thrombus occurring after successful treatment.

### Statistical analyses

Demographic data of the participants were analyzed using percentages and proportions for categorical data, means with standard deviations (SD), or median with interquartile range (IQR) for continuous data. For categorical data, comparisons were performed using the chi-square test or Fisher’s exact test. We used Student`s t-test or Mann Whitney-U test for continuous data. Logistic regression was used to demonstrate the association between clinical variables and outcomes. Odds ratios (OR) with corresponding 95% confidence interval (CI) were reported. The primary outcome was analyzed using the logistic regression for repeated measures. We compared the mRS at initial presentation, 3 months and 6 months. Bonferroni correction was applied. The results were considered statistically significant if the P-value (P) was less than 0.05. All statistical analyses were performed using Stata/IC 16.1 for Windows (StataCorp. 2019. Stata Statistical Software: Release 16. College Station, TX: StataCorp LLC).

## Results

**Fig 1:**
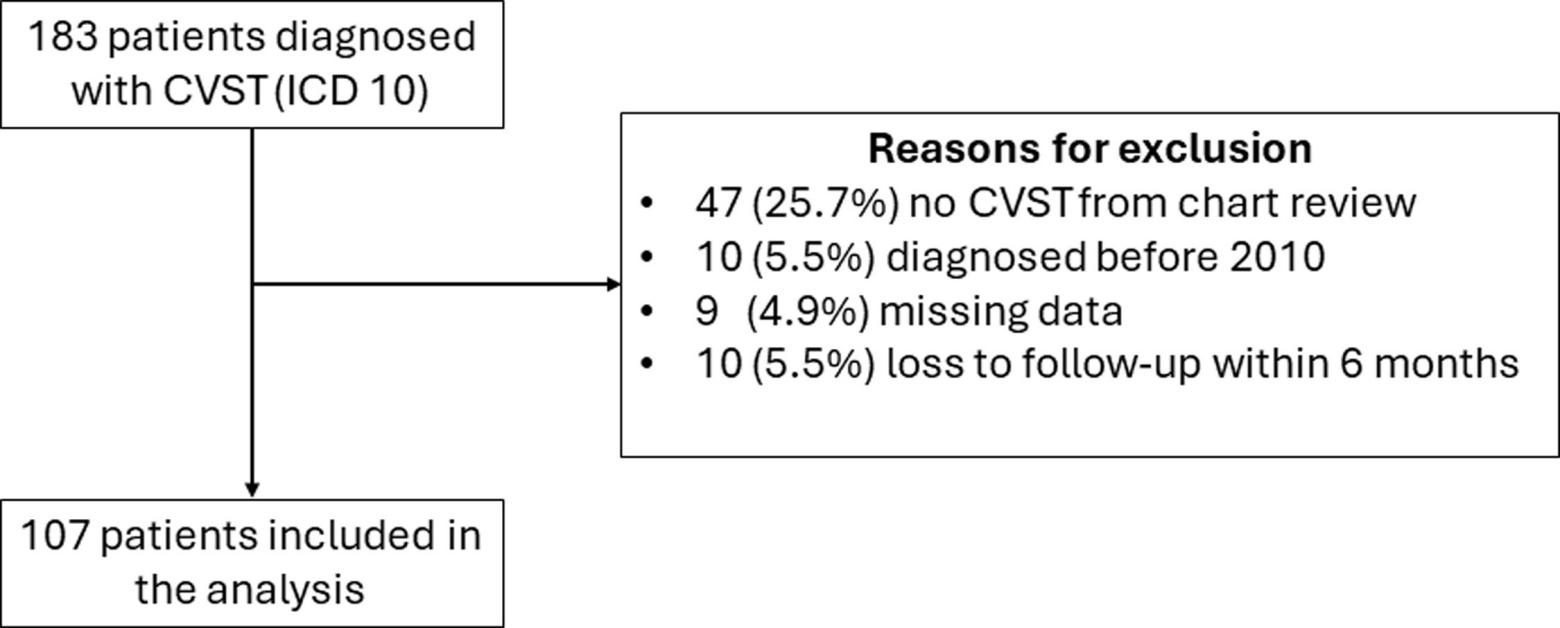
Patient flow diagram.

Of the 183 patients with CVST documented by 10th revision of the International Statistical Classification of Diseases (ICD-10), 47 patients were misdiagnosed and considered to have other diagnoses. Ten patients were diagnosed before the prespecified analytic data period, ten patients were lost to follow-up within six months after treatment, and nine patients had missing data. After exclusion, 107 patients were included in the analysis, as shown in Fig 1. Table 1 demonstrated the baseline demographic data for the study population. The mean age ± SD at diagnosis was 42.7 ± 20.4 years, and 58 patients were female (54.2%). Conventional contrast-enhanced CT brain was the most frequent neuroimaging technique (76.6%), followed by CT venography (27.1%). The most common initial presentation was a headache, which occurred in 80 out of 107 cases (74.8%), followed by altered mental status (33.5%), motor weakness (26.2%), and seizure (23.4%). The contributing factors for CVST was summarized in Table 1. Hormonal therapy was the most common contributing factor, accounting for 25 cases (23.4%), followed by local infection (18.7%) and vascular abnormalities (11.21%), respectively.

**Table 1:** Baseline demographic data.

Characteristics	Total (n = 107)
Age at diagnosis, years [mean ± SD]	42.7 ± 20.4
Female (n, %)	58, 54.2%
Modalities of diagnosis (n, %)	
Conventional CT brain	82, 76.6%
CT venography	29, 27.1%
Conventional MRI brain	10, 9.3%
MR venography	7, 6.5%
Locations (n, %)	
Superior sagittal sinus	58, 54.2%
Sigmoid sinus	55, 51.4%
Transverse with lateral sinus	67, 62.2%
Straight sinus	8, 7.5%
Presenting symptoms (n, %)	
Headache	80, 74.8%
Seizure	25, 23.4%
Alter mental status	36, 33.6%
Motor weakness/ paresis	28, 26.2%
Sensory loss	2, 1.9%
Papilledema	4, 3.7%
Contributing factors (n, %)	
Local infection*	20, 18.7%
Mechanical trauma	9. 8.4%
Vascular abnormalities	12, 11.2%
Myeloproliferative neoplasm	2, 1.9%
Hormonal therapy	25, 23.4%
Neoplasm**	8,7.5%
Idiopathic	34, 31.8%

**Abbreviations:** SD: Standard deviation, IQR: Interquartile range, CT: Computed tomography, MRI: Magnetic resonance imaging

*Local infection refers to meningitis, otitis media, mastoiditis, sinusitis, and head and neck infection.

**Neoplasm refers to all solid and hematologic malignancies regardless of their site.

The median follow-up was 37 months (95% CI 40.3–54.7).

### Anticoagulant treatment

Regarding anticoagulant treatment, 38 patients (35.5%) received unfractionated heparin (UFH), and 66 patients (61.7%) received low-molecular-weight heparin (LMWH). Subsequently, 20 patients (18.7%) switched from UFH to LMWH during the initial phase of treatment. For the long-term treatment, the most common anticoagulant was warfarin (80.4%), followed by DOACs (6.5%). For overall treatment, 103 patients (96.3%) received anticoagulant treatment either acute or long-term treatment. Out of the total cases, 4 (3.7%) did not receive any anticoagulant treatment. Two of them were not given anticoagulants due to the physician’s decision, another one was treated with endovascular intervention without anticoagulant, and the last one was given aspirin instead.

### Neurological outcomes

**Fig 2:**
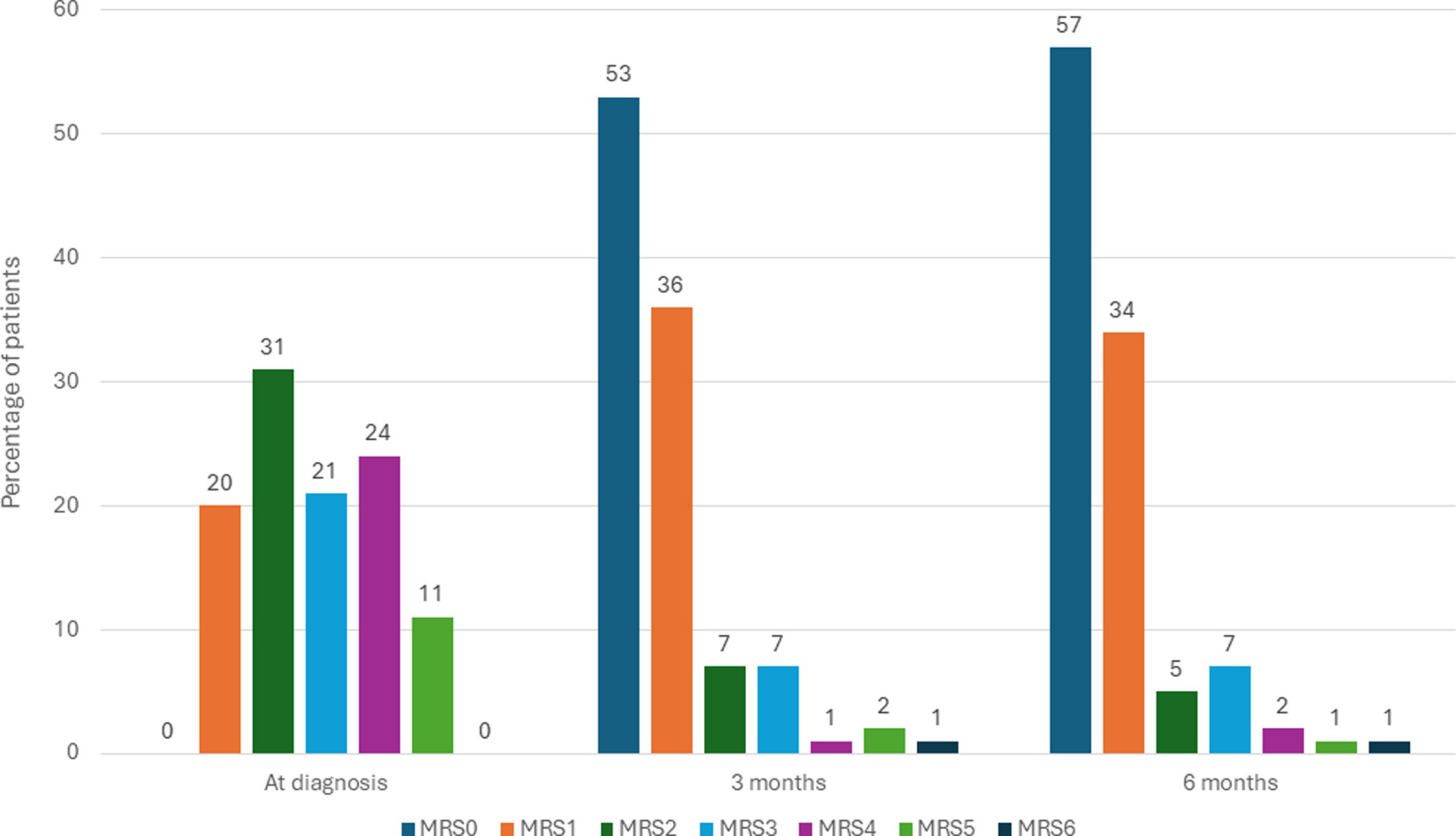
Modified Rankin Scale in patients with CVST at diagnosis, 3 months, and 6 months.

Neurological outcomes measured by mRS were demonstrated in Fig 2. The proportion of patients who were classified as having no disability or no significant disability (mRS < 2) increased from 20 patients (18.7%) at diagnosis to 89 patients (83.2%) at three months and 91 patients (85.1%) at six months after diagnosis. Conditional logistic regression revealed a statistically significant difference in mRS when comparing scores at diagnosis, three months, and six months (P < 0.01). After applying the Bonferroni correction, there was a statistically significant difference when comparing mRS at diagnosis versus three months (P < 0.001) and mRS at diagnosis versus six months (P < 0.001). However, there was no statistically significant difference between mRS at three months and six months (P = 0.525).

No patients were classified as having no residual symptoms (mRS < 1) at diagnosis, but the number increased to 53 patients (49.5%) at three months and 57 patients (53.3%) at six months (P = 0.03 and P = 0.04, respectively).

Table 2 illustrates the association between clinical variables and neurological outcomes at 6 months. The clinical variables were associated with residual neurological symptoms (mRS ≥ 1) included age ≥ 50 years (OR 4.1, 95% CI; 1.7–9.4, P-value 0.001), male sex (OR 3.0, 95% CI; 1.4–6.6, P-value 0.006), the thrombus involvement in deep sinus system (OR 6.1, 95% CI; 1.1–76.9, P-value 0.04), CVT score ≥ 1 (OR 3.1, 95% CI; 1.3–725, P-value 0.014). Patients with active cancer was not associated with residual neurological symptoms as compared to those without, OR 1.2, 95% CI 0.3–4.2, P-value 0.83. The type of anticoagulant was compared, yet no statistically significant difference was found between patients using DOACs compared to warfarin (OR 1.1, 95% CI 0.2–5.2, P-value 0.90). Patients whose CVST was associated with with hormonal therapy were found to have a lower risk of residual neurological symptoms (OR 0.2, 95% CI 0.1–0.6, P-value 0.004).

**Table 2:** The association of clinical variables and residual neurological symptoms measured by modified Rankin Scale ≥ 1 at 6 months.

Variables	Neurological outcome		Logistic regression	
	mRS < 1 n (%)	mRS ≥ 1 n (%)	Odds ratio (95% CI)	P-value
Age				
• < 50 years	45 (65.2)	24 (37.8)	Reference	
• ≥ 50 years	12 (31.6)	26 (68.4)	4.1 (1.7–9.4)	0.001
Sex				
• Female	38 (65.5)	20 (34.5)	Reference	
• Male	19 (38.8)	30 (61.2)	3.0 (1.4–6.6)	0.006
Cancer				
• Absence	52 (53.6)	45 (46.4)	Reference	
• Presence	5 (50.0)	5 (50.0)	1.2 (0.3–4.2)	0.83
Hormonal use				
• Absence	37 (45.1)	45 (54.9)	Reference	
• Presence	20 (80.0)	5 (20.0)	0.2 (0.1–0.6)	0.004
Thrombus at deep sinus				
• Absence	56 (56.6)	43 (43.4)	Reference	
• Presence	1 (12.5)	7 (87.5)	6.1 (1.1–76.9)	0.04
Number of thrombus site(s)				
• Single	20 (47.6)	22 (52.4)	Reference	
• Multiple	37 (56.9)	28 (43.1)	0.7 (0.3–1.5)	0.35
CVT score				
• < 1	23 (71.9)	9 (28.1)	Reference	
• ≥ 1	34 (45.3)	41 (54.7)	3.1 (1.3–7.5)	0.014
Anticoagulant starting time				
• <24 hours	25 (59.5)	17 (40.5)	Reference	
• ≥24 hours	32 (49.2)	33 (50.8)	1.5 (0.7–3.3)	0.309

**Abbreviations:** CI; confidence interval, CTV score; cerebral vein and dural sinus thrombosis risk score, mRS; modified Rankin Scale

### CVT risk score

When classifying patients by CVT risk score, a significantly higher proportion of high-risk patients had an mRS score ≥ one at three months of follow-ups (72.2%) compared to low-risk patients (46.1%) with an OR of 3.0 (95% CI 1.0–9.3, P = 0.05). However, at the six-month follow-up, 61.1% of high-risk patients had mRS ≥ 1, as compared to 43.8% in low-risk patients, OR 2.0 (95% CI 0.7–5.7, P = 0.19). When we classified patients using CVT < 1 versus ≥ 1, patients with CVT score ≥ 1 were associated with residual neurological outcome (Table 2).

### Mortality outcome

All-cause mortality was observed in three of 107 patients (2.8%). There was one patient who died after having major gastrointestinal bleeding while receiving LMWH. The other two patients died from infectious causes.

### Bleeding complications

During the follow-up period, 14 patients (13.1%) experienced bleeding complications. Out of these, seven patients had major bleeding, while the rest had minor bleeding. Of the seven patients with minor bleeding, four patients had gastrointestinal clinically relevant non-major bleeding.. Of patients who had major bleeding, five patients had intracranial bleeding and two patients had extracranial bleeding.

The presence of malignancy at diagnosis (OR 5.8, 95% CI 1.4–24.1; P-value 0.016) and thrombus involvement in deep sinus (OR 4.8, 95% CI 1.0–22.9; P-value 0.049) were an independent risk factor of bleeding There was no statistical difference in terms of bleeding in patients using a different type of anticoagulant, sex and age as shown in Table 3.

**Table 3:** The association of clinical variables and bleeding symptoms at 6 months.

Variables	Bleeding outcome		Logistic regression	
	Absence n (%)	Presence n (%)	Odds ratio (95% CI)	P-value
Age				
• < 50 years	61 (88.4)	8 (11.6)	Reference	
• ≥ 50 years	32 (84.2)	6 (15.8)	1.4 (0.5–4.5)	0.539
Sex				
• Female	48 (82.8)	10 (17.2)	Reference	
• Male	45 (91.8)	4 (8.2)	0.4 (0.1–1.5)	0.174
Cancer				
• Absence	87 (89.7)	10 (10.3)	Reference	
• Presence	6 (60.0)	4 (40.0)	5.8 (1.4–24.1)	0.016
Hormonal use				
• Absence	72 (87.8)	10 (12.2)	Reference	
• Presence	21 (84.0)	4 (16.0)	1.4 (0.4–4.8)	0.622
Thrombus at deep sinus				
• Absence	88 (88.9)	11 (11.1)	Reference	
• Presence	5 (62.5)	3 (37.5)	4.8 (1.0–22.9)	0.049
Number of thrombus site(s)				
• Single	40 (95.2)	2 (4.8)	Reference	
• Multiple	53 (81.5)	12 (18.5)	4.5 (1.0–21.4)	0.057
CVT score				
• < 1	27 (84.4)	5 (15.6)	Reference	
• ≥ 1	66 (88)	9 (12.0)	0.7 (0.2–2.4)	0.612
Type of anticoagulant				
• Warfarin	66 (84.8)	12 (15.2)	Reference	
• DOACs	6 (84.9)	1 (14.3)	0.9 (0.1–8.4)	0.949

**Abbreviations:** CI; confidence interval, CTV score; cerebral vein and dural sinus thrombosis risk score, mRS; modified Rankin Scale, DOAC; direct oral anticoagulant

### Duration of anticoagulant and recurrence

Fifty-four patients (50.5%) had discontinued anticoagulants, with a median duration of 31 months, compared to 53 patients (49.5%) who continued anticoagulation for a median duration of 43 months. Patients who presented with isolated headaches were associated with anticoagulant discontinuation (OR 6.6, 95% CI 2.8–14.6, P = 0.01). Hormonal therapy was associated with anticoagulant discontinuation (OR 2.7, 95% CI 1.1–7.0; P = 0.04).

Four patients (3.7%) experienced recurrent CVST. Two were taking warfarin before recurrence, but at subtherapeutic levels of International Normalized Ratio (INR). The other two patients, who had the vascular risk factor of dural arteriovenous fistula, experienced recurrence after discontinuing anticoagulant therapy for three and seven years, respectively. There was no association between anticoagulant discontinuation and recurrence (HR 0.7, 95% CI 0.1–5.1; P = 0.74).

### Recanalization

A total of 88 patients (82.2%) underwent follow-up imaging studies as physician decision after treatment around one year. Of these, 55 patients (62.5%) had either partial or complete recanalization at one year. There was no significant difference in anticoagulant discontinuation between patients who had recanalization and those who did not (P = 0.90). In addition, there was no significant difference in the recurrence rate between patients who had recanalization (3.6%) and those who did not (6.1%), P = 0.60.

## Discussion

The current study demonstrated a significant improvement in neurological outcomes as measured by mRS at three months and six months after the diagnosis of CVST.

It is important to note that the population of this study had slightly female predominance, and the most common contribution factor was hormonal therapy (23% of patients). This finding differed from the large ISCVT cohort of 624 CVST patients, which reported that 74.5% of patients were female, and 54% of patients had CVST secondary to oral contraceptive pills [16]. In addition, the patients in this present study were older than those in the ISCVT study (mean age of 42.7 years compared to 39.1 years). These differences may have implications for the outcomes. Therefore, the interpretation of our findings should be interpreted with caution.

While headache was the most common presentation of CVST, one-third of patients in this study had altered mental status, resulting in a majority of patients having a high mRS score at presentation. However, we observed a significantly increased proportion of patients who had no disability or no significant disability (mRS < 2) from 18.7% at diagnosis to 83.2% at three months and 85.1% at six months after the initial treatment of CVST. In comparison, the ISCVT study assessed neurological outcomes and found that at the end of follow-up (median 16 months), 79.1% of patients had no disability or no significant disability. A more recent study by Triquenot Bagan et al., which reported on 231 CVST patients from the French registry, found that 181 patients (78.4%) had an mRS score < 2 at a median follow-up of 11.9 months [21]. These findings suggest that CVST patients have a low rate of remaining disability associated with the condition.

Concerning anticoagulant treatment, we found no statistically significant difference in clinical outcomes among patients using different types of anticoagulation therapy. Long-term anticoagulation with warfarin was the most common treatment option. However, a small number of patients received DOACs, which may be contributed to the fact that the majority of patients who were enrolled before the DOACs era showed benefits for the treatment of CVST [22]. Previous studies revealed that LMWH was associated with a lower numerical risk of in-hospital mortality [23] and a slightly lower rate of intracranial hemorrhage [24]. When selecting an anticoagulant, the choice should be based on clinical presentation and patients’ preferences.

There are several risk factors predicting poor neurological outcomes, including altered mental status, malignancy, coma, thrombosis of the deep venous system, male gender, and intracranial hemorrhage at presentation [25]. This study found that patients who developed CVST secondary to hormonal therapy were associated with better neurological outcomes, in terms of no residual symptoms. The odds ratio of 4.9 indicates that patients with hormonal factors were almost five times more likely to have no residual symptoms compared to those without. In addition, when we classified patients according to the CVT risk score, those in the high-risk category had a three-fold increased risk of having a neurological residual disability. However, while there were numerically higher proportions of patients with disability in the high-risk group, the CVT risk score was not statistically associated with an increased risk of neurological disability at six months. This study emphasized the importance of assessing patients at the initial diagnosis to facilitate close monitoring and plan appropriate treatment, to improve neurological outcomes.

In this study, 13% of patients experienced bleeding, of which 7% were major bleeds. The gastrointestinal tract was the most common site of major bleeding. In usual sites of venous thromboembolism, the risk of major bleeding with anticoagulant therapy is reported to be 1.3–2.2 per 100 patient-year [26, 27]. In a recent clinical trial comparing dabigatran to warfarin in patients with CVST for six months, major bleeding was found in 1.7% of the dabigatran group and 3.3% in the warfarin group [6]. Notably, the trial excluded patients with malignancy. The high number of major bleeding in this study may reflect real-world data, where many CVST patients have active malignancies. In this present study, patients with active malignancy had a 4.9 times higher risk of major bleeding, according to our findings. These findings suggested providing close monitoring of the patients with active malignancy, minimizing the risk of bleeding complications during anticoagulant therapy for CVST.

Currently, there was no conclusive evidence on the optimal duration of anticoagulant treatment for CVST [28]. Our study found that patients with hormonal-related CVST had a 2.7 times higher chance of anticoagulant discontinuation compared to those without hormone-related CVST. Furthermore, our results showed that the risk of recurrence did not differ significantly between patients who discontinued anticoagulant therapy and those who continued. These findings suggest that anticoagulant discontinuation may be safe in selected patients with CVST.

The study had several limitations to be addressed. First, the factors that may have contributed to the cause of CVST were not systematically investigated. Inherited thrombophilia and antiphospholipid antibodies screening were performed upon the attending physician’s request. Second, the study population included patients treated since 2010, when warfarin was the standard oral anticoagulant therapy. As a result, there were only a small number of patients treated with DOACs, which precluded analysis of outcomes among patients prescribed different oral anticoagulants. Third, the CVST patients secondary to vaccine-induced thrombotic thrombocytopenia (VITT) were not included in the study. Therefore, the generalizability of the study findings to patients with CVST caused by COVID-19 vaccination may be limited. Lastly, we were unable to determine the appropriate duration of anticoagulant therapy. However, we observed that patients who discontinued anticoagulants at some point had a median treatment duration of 31 months.

## Conclusions

A significant improvement in neurological outcomes was observed at 3 and 6 months after diagnosis. Older age, male sex, thrombus involvement in deep sinus system were associated with residual neurological symptoms. In certain patients, anticoagulant therapy can be safely discontinued, especially in those with provoked CVST due to hormonal therapy.
